# Cognitive function and fatigue before and after transsphenoidal surgery in patients with pituitary adenoma: a prospective study

**DOI:** 10.1007/s11102-025-01527-y

**Published:** 2025-05-06

**Authors:** David Krabbe, Tamar Abzhandadze, Thomas Skoglund, Tobias Hallén, Daniel S. Olsson, Victor Hantelius, Oskar Ragnarsson, Sofie Jakobsson, Gudmundur Johannsson, Katharina S. Sunnerhagen

**Affiliations:** 1 Department of Rehabilitation Medicine, Sahlgrenska University Hospital, Gothenburg, 400 43 Sweden; 2 Department of Clinical Neuroscience, Institute of Neuroscience and Physiology, Sahlgrenska Academy, University of Gothenburg, Gothenburg, 405 30 Sweden; 3 Department of Occupational Therapy and Physiotherapy, Sahlgrenska University Hospital, Gothenburg, 413 45 Sweden; 4 Department of Neurosurgery, Sahlgrenska University Hospital, Gothenburg, 413 45 Sweden; 5 Department of Endocrinology, Sahlgrenska University Hospital, Gothenburg, 413 45 Sweden; 6 Department of Internal Medicine and Clinical Nutrition, Institute of Medicine, Sahlgrenska Academy, University of Gothenburg, Gothenburg, 405 30 Sweden; 7 Wallenberg Centre for Molecular and Translational Medicine, Institute of Medicine, Sahlgrenska Academy, University of Gothenburg, Gothenburg, 405 30 Sweden; 8 Centre for Person-Centred Care (GPCC), University of Gothenburg, Gothenburg, 405 30 Sweden; 9 Institute of Health and Care Sciences, Sahlgrenska Academy, University of Gothenburg, Gothenburg, 405 30 Sweden; 10 Department of Clinical Neuroscience, SU/Sahlgrenska, Blå stråket 7, plan 3, Göteborg, SE-413 45 Sweden

**Keywords:** Pituitary neoplasms, Transsphenoidal surgery, Neuropsychological tests, Fatigue, Cognitive function

## Abstract

**Purpose:**

The aim of this prospective longitudinal study was to evaluate cognitive function and fatigue before and 12 months after transsphenoidal surgery (TSS) for a pituitary adenoma.

**Methods:**

This study was part of the Gothenburg Pituitary Tumour Study, which consecutively includes patients undergoing TSS at Sahlgrenska University Hospital. Adult patients with a pituitary adenoma were recruited between October 2016 and May 2021. Cognitive function and fatigue were evaluated using the Repeatable Battery for the Assessment of Neuropsychological Status (RBANS) and the Multidimensional Fatigue Inventory (MFI-20). Paired comparisons were made for total and subscale scores before and after TSS. Based on normative data, individual scores were classified into one of three categories for symptom severity (normal, moderate, or severe) before and after TSS.

**Results:**

Fifty-nine patients (31 females) were included. Among them, 42 had non-functioning pituitary adenomas (NFPA) and 17 had a functioning pituitary adenoma. There were no differences in RBANS total or domain indices before and 12 months after surgery except for the attention index which improved. Total MFI-20 and all subscale scores improved. The improvement was more pronounced in patients with functioning pituitary adenoma, who reported worse fatigue before surgery compared to patients with NFPA. Individual differences between pre- and postoperative scores that also changed category of symptom severity were seen for 37% of all patients regarding cognition and for 35% regarding fatigue. Improvements accounted for the majority of these changes.

**Conclusion:**

Cognitive function remained largely unchanged from before to 12 months after TSS, while self-reported fatigue improved.

**Supplementary Information:**

The online version contains supplementary material available at 10.1007/s11102-025-01527-y.

## Introduction

Pituitary adenomas, although classified as benign tumors, can impact health by being functionally active and by causing pituitary hormone deficiencies and/or neurological symptoms including visual impairment due to their size and impact on surrounding structures [1]. Such consequences have been found to significantly impact both physical and mental functioning, as reported by patients prior to primary tumor treatment [2].

The management of symptomatic pituitary adenomas typically involves surgical intervention, medical therapy, and occasionally radiotherapy [3]. Transsphenoidal surgery (TSS) is, in general, the treatment of choice for most types of pituitary adenomas [4]. This procedure is performed via an endonasal route, which may minimize manipulation of adjacent brain structures, though some impact on neuronal structure cannot be entirely excluded. Hypothalamic injuries, for example, may affect regulation of sleep-wake cycles [5] and may have a negative impact on optimal cognitive functioning [6]. Treatment can effectively alleviate initial symptoms by controlling hormonal excess, addressing pituitary hormone deficiencies, and relieving mass effects [5].

Nonetheless, the tumor and its treatment may have long-standing consequences such as pituitary deficiency and hypothalamic dysfunction [5]. Patients usually require life-long care for tumor surveillance and endocrine replacement therapies due to pituitary hormone deficiency [7, 8]. Long-term follow-up studies after treatment for pituitary adenoma have indicated that pituitary disease has a negative effect on wellbeing and general health [9, 10]. Cognitive problems and fatigue are frequently reported complaints by patients [11, 12].

In a review on the impact of TSS on cognitive function [13], it was concluded that TSS may affect cognition, although the literature was limited and inconsistent. Regarding fatigue, vitality has been shown to improve at 3 and 6 months after TSS compared to preoperative status [14, 15]. In a recent study [16], we found that variability in cognition was mainly explained by sex, educational level, and fatigue rather than previous TSS.

Nevertheless, uncertainty still exists to what extent cognitive function and fatigue are altered after TSS. The aim of this study was to evaluate cognitive function and fatigue before and 12 months after TSS in the same patients.

## Materials and methods

### Design

This prospective longitudinal study was part of the Gothenburg Pituitary Tumour Study (GoPT-study; registration number 161671), which consecutively includes patients undergoing TSS at Sahlgrenska University Hospital, the primary neurosurgical center in western Sweden serving ~ 1.9 million people. Evaluation of cognitive function and fatigue is part of the data collection of the GoPT-study. Detailed information about the GoPT-study has been published elsewhere [17]. Patients were recruited to the present study between October 2016 and May 2021. A within-subject design was used, with change in cognitive function and fatigue being analyzed based on the results combined and individually by norm-based symptom severity.

### Participants

Out of 200 patients scheduled for TSS during the study period, 191 were enrolled in the GoPT-study. Patients underwent TSS for pituitary adenoma or other types of pituitary tumors, either as primary surgery or reoperation due to progression of residual tumor.

**Fig. 1:**
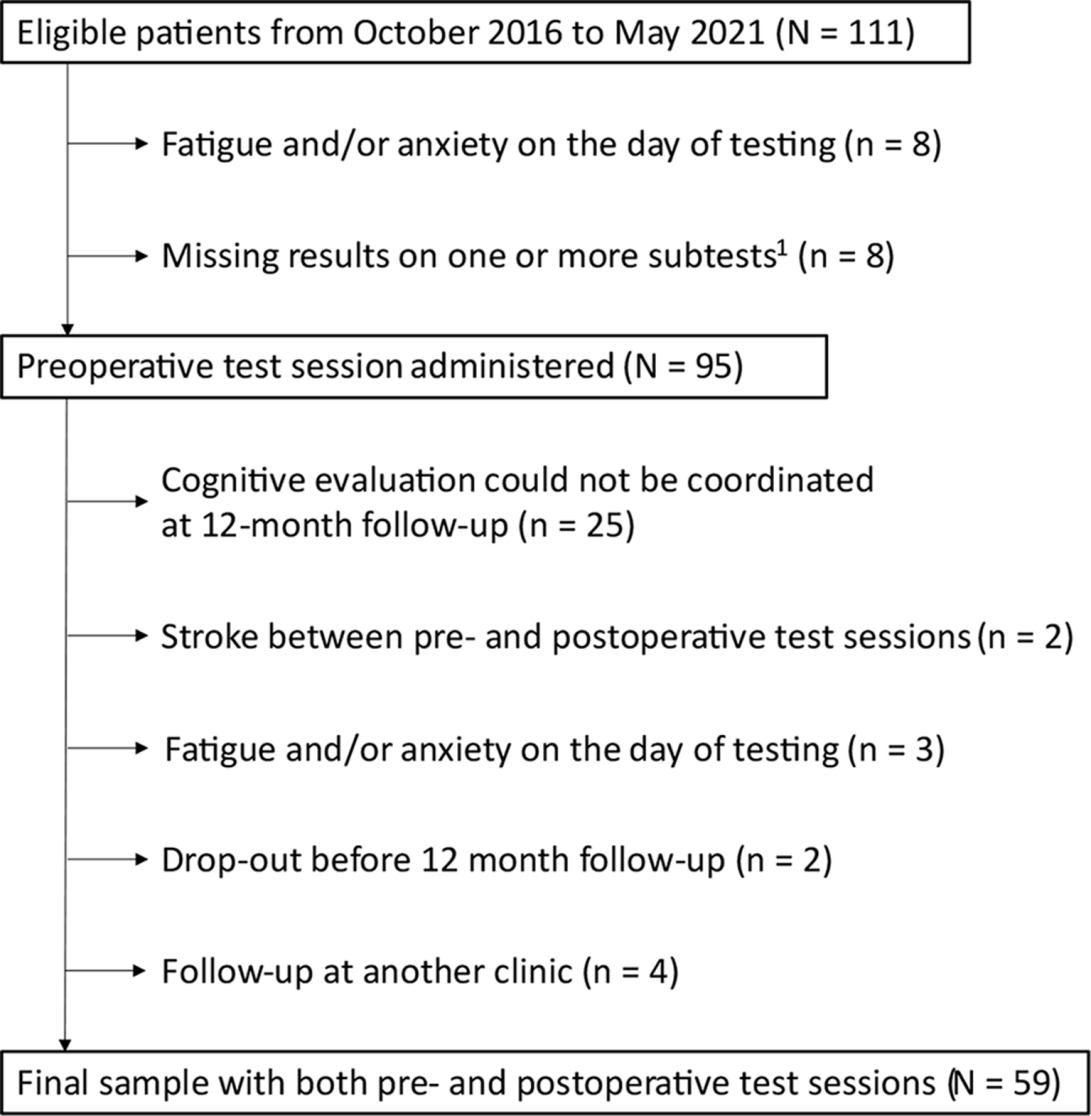
Flow chart. ^1^ Missing results due to patients having impaired sight (*n* = 2), difficulties in writing due to a venous catheterization (*n* = 1), unaccustomed to the Swedish language (*n* = 4), and lack of one of the test forms at the time for testing (*n* = 1)

Inclusion criteria for the present study were a confirmed diagnosis of pituitary adenoma, age ≥ 18 years, and informed consent. Stroke in the past medical history was an exclusion criterion. Preoperative cognitive evaluation could be coordinated for 111 patients. After further exclusions (Fig. 1), a final sample of 59 patients who completed cognitive evaluation both pre- and postoperatively remained. The patients included in the final sample did not differ significantly from other patients scheduled for TSS and participating in the GoPT-study with respect to age (*p* = 0.251). However, the proportion of women in the final sample was higher (53% vs. 35%; *p* = 0.021).

### Ethics

Approval from the regional ethical review board in Gothenburg, Sweden, was obtained (Dnr: 387 − 15, T682-16). This study was conducted in accordance with the Declaration of Helsinki, ensuring adherence to ethical principles for medical research involving human subjects. All participants provided oral and written informed consent prior to their inclusion in the study.

### Measurements

#### Hormonal status

Tumor type was determined based on clinical, histopathological, and biochemical evaluation. An endocrinologist evaluated pre- and postoperative anterior pituitary function through biochemical analyses and clinical evaluation, and assessed the need for and adequacy of hormone replacement therapy at the time of the evaluation of fatigue and cognition. Biochemical analyses for all hormonal axes were performed before surgery, within 1 week after surgery, and at postsurgical follow-up at 3–6 months except testing for growth hormone deficiency, which was first done 9–12 months after surgery. Arginine vasopressin deficiency was considered chronic if desmopressin replacement therapy was ongoing 1 year after surgery.

#### Cognition

Cognitive function was evaluated using the Swedish version of the Repeatable Battery for the Assessment of Neuropsychological Status (RBANS) [18]. RBANS has reference data from 454 individuals 20–89 years of age from the Scandinavian normal population. The selection of the normative sample was based on official statistics from these countries and was aimed to correspond to the age distribution and educational level of the normal population. Exclusion criteria were, for example, medical or psychiatric conditions that might affect cognition and lack of sufficient language skills. RBANS includes 12 subtests, each contributing to a cognitive domain: immediate memory (list learning, story memory), visuospatial/constructional (figure copy, line orientation), language (picture naming, semantic fluency), attention (digit span, coding), and delayed memory (list recall, list recognition, story recall, figure recall). The index scores of the domains are corrected for age and can be converted into a total index score. The mean for domain and total indices is 100 and standard deviation (SD) is 15. To minimize practice effects of repeated assessment, two parallel Swedish versions (A and B) with different content are available. Administration time is approximately 25 min. In this study impairments were classified based on procedures described previously [19, 20] and combination of domain index scores. Impairments were classified as mild to moderate in case of two scores 1.5 SD below the mean or one score 2 SD below the mean, and as severe in case of three or more scores 1.5 SD below the mean or two or more scores 2 SD below the mean.

#### Fatigue

Fatigue was evaluated using the Swedish version of the Multidimensional Fatigue Inventory (MFI-20), which includes five subscales: general fatigue, physical fatigue, mental fatigue, reduced activity, and reduced motivation) [21]. Answers are given on a five-point scale and the range for each subscale is 4–20. Higher values indicate higher levels of fatigue. Although MFI-20 was originally developed to measure the different dimensions of fatigue, use of the total score (range 20–100) has also been recommended [22]. Cutoff levels based on the 75th and 90th percentiles of the general fatigue subscale score in a German general population sample [23] have previously been used for moderate and severe fatigue, respectively [24]. Population-based normative data on the general fatigue subscale was provided from the MONICA Study [25] by personal communication (M. Eliasson). From this, the 75th and 90th percentiles could be produced stratified by sex and 10-year age groups. In the present study, general fatigue scores above the 75th percentile were categorized as moderate fatigue and scores above the 90th percentile as severe fatigue.

### Procedure

RBANS was administered in a quiet office at the Department of Neurosurgery the day before surgery and at the Department of Endocrinology at 1-year follow-up (both departments are part of the same hospital). All test administrations were made by the same psychologist (DK). For 20 patients, RBANS version A was used before surgery and version B at 1-year follow-up, and for 39 patients vice versa. Mean and median interval between test administrations was 370 days (range 327–454; SD 24). A research nurse administered MFI-20 and the patients completed it on paper or on a digital tablet. All patients underwent endoscopic TSS. All surgeries were performed by an ear, nose, and throat surgeon and a neurosurgeon together.

### Statistics

Sample size calculations showed that minimum sample size to detect a medium effect size in change for cognition was 54 (Cohen’s d 0.5, α error probability 0.05, power 0.95, calculated using G-Power 3.1).

Normality was examined using the Shapiro-Wilk test. Between-group comparisons were made with Mann-Whitney *U*-test for variables with skewed distributions and Pearson’s χ^2^ test for binary variables. Within-group comparisons were made with paired *t*-test for variables with normally distributed data and Wilcoxon signed ranks test for variables with skewed distributions. Comparisons with reference population data were made with one-sample *t*-test for variables with normally distributed data and one-sample Wilcoxon signed ranks test for variables with skewed distributions.

For cognition and fatigue, change was calculated by subtracting preoperative from postoperative score. Effect sizes were estimated using r, with the z-value divided by the square root of number of observations [26] or Cohen’s d. Thresholds for small, medium, and large effect sizes were 0.1, 0.3, and 0.5 for r and 0.2, 0.5, and 0.8 for d [27]. Spearman’s correlation was used for a subsequent analysis of the relation between change in cognition and change in fatigue. In a subgroup analysis, within-group comparisons were made for the groups with nonfunctioning pituitary adenoma (NFPA) and functioning pituitary adenoma separately.

On two occasions, one MFI-20 item was left unanswered. These missing values were replaced with mean imputation, i.e. using the mean of the answered items of that subscale. For seven patients (4 females, 3 males), MFI-20 change could not be calculated due to response only once. SPSS version 27 (IBM Corp., Armonk, NY) was used for statistical analyses. Sankey diagrams were made using SankeyMATIC. All statistical tests were two tailed. The alpha level was set at 5% for statistical significance.

## Results

Fifty-nine patients with complete data before and after surgery were included in the final analysis (Fig. 1). Data concerning demographics, diagnosis, anterior pituitary hormone deficiency, arginine vasopressin deficiency, and hormone replacement are presented in Table 1. The majority of patients had at least one known pituitary deficiency both before surgery (*n* = 33) and 12 months after (*n* = 37). Forty-two patients had NFPAs and 17 had functioning pituitary adenomas. Biochemical control at 12 months after surgery was achieved for all patients with Cushing’s disease (*n* = 5) and for seven out of nine patients with acromegaly.

**Table 1:** Demographics, diagnoses, hormone deficiencies, and treatments

	All patients (*N* = 59)	
Sex, n (%)		
Female	31 (52.5)	
Male	28 (47.5)	
Age in years at surgery, mean (SD)	57.3 (14.3)	
Years of education, mean (SD)	13.3 (2.9)	
Diagnosis, n (%)		
Non-functioning pituitary adenoma	42 (71.2)	
Acromegaly	9 (15.3)	
Cushing’s disease	5 (8.5)	
Prolactinoma	2 (3.4)	
Thyrotropinoma	1 (1.7)	
Chiasmal compression, n (%)		
No	18 (31)	
Yes	41 (69)	
Type of surgery, n (%)		
Primary	41 (69)	
Reoperation^a^	18 (31)	
	Presurgery	12 months postsurgery
Cortisol, n (%)		
Deficiency and replaced	12 (20.3)	19 (32.2)^b^
Thyroid hormone, n (%)		
Central hypothyroidism	19 (32.2)	27 (45.8)
Primary hypothyroidism	5 (8.5)	5 (8.5)
Replacement	24 (40.7)	31 (52.5)
Sex steroids, premenopausal women (*n* = 11), n (%)		
Deficiency	1 (9.1)	1 (9.1)
Unable to verify^c^	3 (27.3)	3 (27.3)
Replacement^d^	4 (36.4)	4 (36.4)
Sex steroids, postmenopausal women (*n* = 20), n (%)		
Hormone replacement therapy	0	0
Sex steroids, men, n (%)		
Deficiency	17 (60.7)	16 (57.1)
Replacement, testosterone	8 (28.6)	13 (46.4)
Growth hormone, n (%)		
Deficiency^e^	15 (25.4)	26 (44.1)
Not investigated	32 (54.2)	18 (30.5)
Replacement	13 (22.0)	22 (37.3)
Arginine vasopressin, n (%)		
Deficiency and replaced	5 (8.5)	12 (20.3)

^a^ Due to recurrence or progression of a residual pituitary adenoma

^b^ Including one participant with partial deficiency and glucocorticoid replacement as needed

^c^ Deficiency uncertain due to other medication containing estrogen

^d^ Estrogen or testosterone replacement or other medication containing estrogen

^e^ Established by provocative test or low insulin-like growth factor 1 and three other deficiencies

*Abbreviation*: SD, standard deviation

### Cognition

No differences were observed for RBANS total or domain indices comparing before and 12 months after surgery except for a significantly improved attention index postoperatively (Table 2). This change had a medium effect size (d = 0.51, *p* < 0.001) and was due to improvements primarily in the scores of one of the contributing subtests (coding). RBANS total index mean before and 12 months after surgery were significantly lower in comparison with the mean of the reference population (*p* < 0.001). No difference was observed for change in RBANS total index comparing patients with and without preoperative chiasmal compression (*p* = 0.656). Change in RBANS total index was − 3.2 (*p* = 0.497, *n* = 11) for patients who acquired cortisol deficiency after surgery and 2.6 (*p* = 0.067, *n* = 48) for those who did not, but the sample size did not allow for reliable study of this numerical difference. RBANS total index change did not differ significantly between subgroups (*p* = 0.262).

**Table 2:** RBANS and MFI-20 total and subscale scores presurgery and 12 months postsurgery

	Mean (SD)	12 months postsurgery	Difference	*p*	d	*r*
	Presurgery					
RBANS index (*n* = 59)						
Total	87.3 (16.6)	88.8 (16.8)	1.6 (11.0)	0.282	0.14	
Immediate memory	91.3 (16.9)	91.1 (17.8)	–0.2 (13.9)	0.896	–0.02	
Visuospatial	88.9 (12.0)	89.8 (12.2)	0.8 (11.9)	0.587	0.07	
Language	98.6 (19.7)	96.1 (17.5)	–2.4 (16.2)	0.256	–0.15	
Attention	89.8 (15.2)	95.1 (15.6)	5.3 (10.4)	< 0.001	0.51	
Delayed memory	88.6 (16.7)	90.6 (16.5)	2.0 (12.5)	0.222	0.16	
MFI-20 (*n* = 52)						
Total	58.1 (23.4)	50.0 (22.4)	–8.1 (18.3)	0.005		–0.28
General fatigue	13.2 (5.5)	11.5 (5.5)	–1.8 (4.4)	0.003		–0.29
Physical fatigue	12.6 (5.7)	10.8 (5.2)	–1.8 (4.9)	0.017		–0.23
Mental fatigue	10.9 (4.9)	9.7 (5.4)	–1.3 (3.4)	0.019		–0.23
Reduced activity	12.2 (5.5)	10.5 (5.4)	–1.6 (4.8)	0.040		–0.20
Reduced motivation	9.1 (4.2)	7.5 (3.9)	–1.6 (4.0)	0.006		–0.27

Note. Paired samples *t*-tests and Cohen’s effect size (d) were used for comparisons of RBANS indices and Wilcoxon signed ranks tests and effect size r for MFI-20 total and subscale scores

*Abbreviations*: MFI-20, Multidimensional Fatigue Inventory-20; RBANS, Repeatable Battery for the Assessment of Neuropsychological Status; SD, standard deviation

**Fig. 2:**
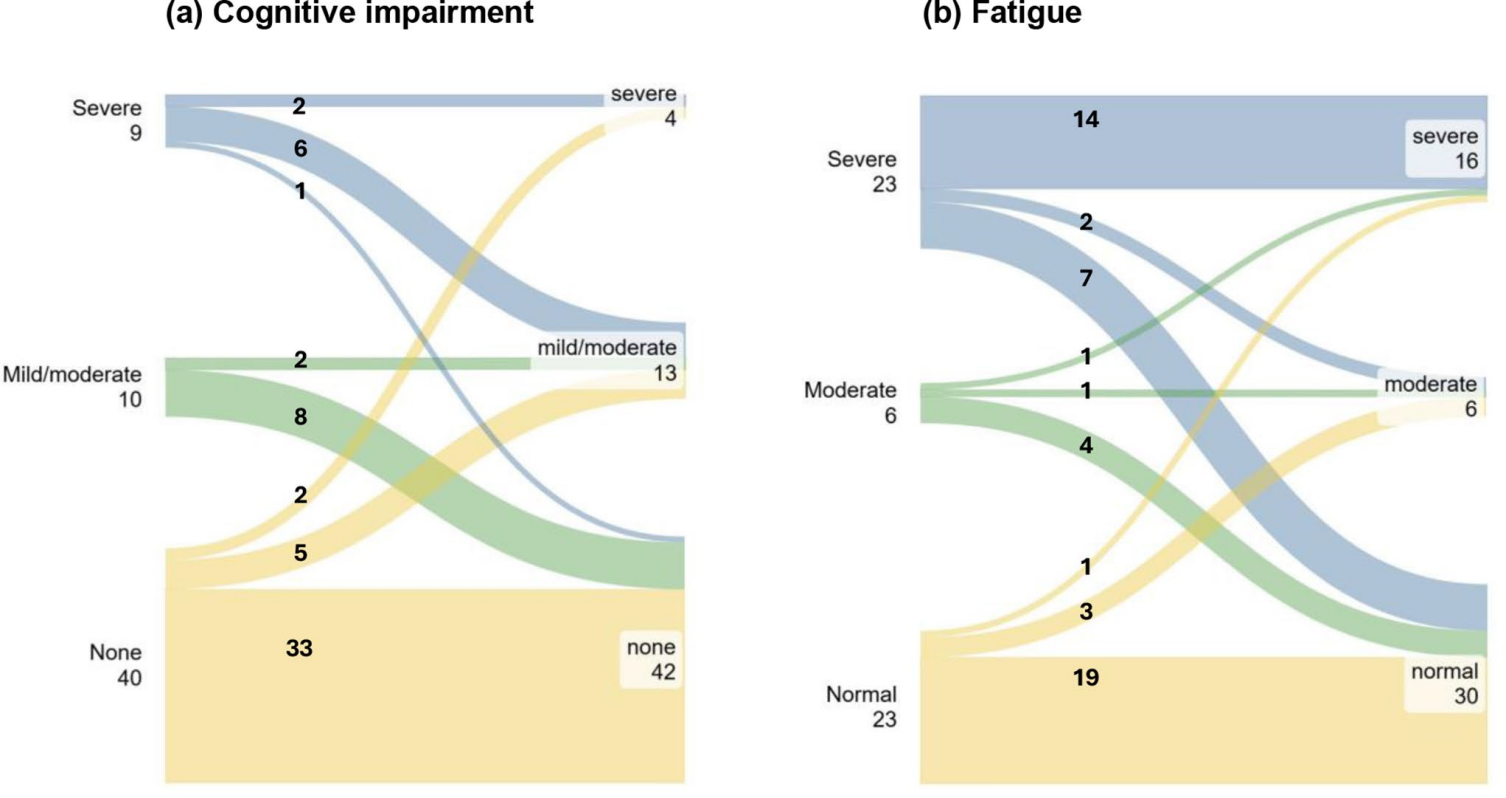
Level of symptoms before and 12 months after TSS and individual change for (**a**) cognition based on RBANS test results and (**b**) for fatigue based on the general fatigue subscale of MFI-20. The procedures for classification of level of cognitive impairment and fatigue are described in the Methods. For MFI-20, the age range of the reference population was 25–74 years. The age of patients was outside this range in seven patients before surgery and eight patients 12 months after surgery. In these cases, results were related to the closest 10-year age group (the 25–34 year group or the 65–74 year group). Abbreviations: RBANS, Repeatable Battery for the Assessment of Neuropsychological Status; MFI-20, Multidimensional Fatigue Inventory-20; TSS, transsphenoidal surgery

Figure 2a illustrates changes in symptom severity for cognition based on RBANS indices from before to 12 months after surgery. Before surgery, nine patients had severe impairment, 10 had mild/moderate impairment, and 40 had no impairment. After surgery, four patients had severe impairment, 13 had mild/moderate impairment, and 42 had no impairment. Seven patients demonstrated a trajectory indicating worsening of symptoms (of these, 3 developed a new anterior pituitary hormone deficiency, 1 had a pituitary hormone deficiency that resolved, and 2 developed a new arginine vasopressin deficiency). Conversely, 15 patients demonstrated a trajectory indicating improvement.

### Fatigue

The MFI-20 total score and all subscale scores decreased after surgery, exhibiting small effect sizes (Table 2). Median scores for the general fatigue subscale were significantly higher in comparison with the median of the reference population, both before (*p* < 0.001) and 12 months after surgery (*p* = 0.041). Females had significantly higher scores than males on the general fatigue subscale before surgery (*p* = 0.030). The effect size of the decrease in general fatigue subscale score after surgery was medium for females (*p* = 0.026, *r*=-0.30) and small for males (*p* = 0.058, *r*=-0.27). No difference was observed for change in MFI-20 total score comparing patients with and without preoperative chiasmal compression (*p* = 0.235). Change in MFI-20 total score was − 6.9 (*p* = 0.401, *n* = 9) for patients who acquired cortisol deficiency after surgery and − 8.3 (*p* = 0.006, *n* = 43) for those who did not. This change did not differ significantly between subgroups (*p* = 0.594).

Figure 2b illustrates changes in fatigue severity based on the general fatigue subscale of MFI-20 from before to 12 months after surgery. Before surgery, 23 patients had severe fatigue, six had moderate fatigue, and 23 had no fatigue. After surgery, 16 patients had severe fatigue, six had moderate fatigue, and 30 had no fatigue. Five patients demonstrated a trajectory indicating worsening of symptoms (of these, three had new anterior pituitary hormone deficiencies, one had deficiencies that resolved, and one had new arginine vasopressin deficiency). Conversely, 13 patients demonstrated a trajectory indicating improvement.

### Cognition related to fatigue

There was no significant correlation between change in RBANS total score and change in MFI-20 total score (r_s_ = − 0.27, *p* = 0.055).

### Subgroup analysis of cognition and fatigue in patients with non-functioning and functioning adenomas

Improvement in the attention index was significant for patients with NFPA but not for those with a functioning pituitary adenoma (Supplementary Fig. 1). No additional significant differences across other RBANS indices were found. Individual change in category of cognitive impairment was seen for 17 of 42 patients with NFPA (40%; 12 to less and 5 to more symptoms) and five of 17 patients with functioning pituitary adenoma (29%; 3 to less and 2 to more symptoms), Supplementary Fig. 3a.

Regarding change in fatigue, significant improvement for patients with NFPA was seen for the reduced motivation subscale (with a small effect size; Supplementary Fig. 2). For the group with functioning pituitary adenoma, significant improvements were seen for total score and physical fatigue (with medium effect sizes) and general fatigue (with a large effect size). Individual change in category of fatigue severity was seen for 13 of 39 patients with NFPA (33%; 9 to less and 4 to more symptoms) and five of 13 patients with functioning pituitary adenoma (38%; 4 to less and 1 to more symptoms), Supplementary Fig. 3b.

## Discussion

In this prospective longitudinal study, overall comparisons showed largely unchanged cognitive function and improved self-reported fatigue from before to 12 months after TSS. For both cognition and fatigue, individual comparisons showed stable symptom severity in just under two-thirds of the patients.

Although cognitive function was largely unchanged, the attention domain showed improvement after 12 months. Our results are in line with previous findings that surgery does not worsen cognitive impairments [28–31]. Before surgery, approximately one-third of patients met the criteria for either mild/moderate or severe cognitive impairment and this proportion remained similar after surgery. However, it is noteworthy that the patients who met the criteria for cognitive impairment before surgery were not necessarily the same as those who did after surgery. Thus, individual change occurred, reflecting previous findings of markedly different individual courses that do not surface in group level comparisons [32].

Fatigue was reduced 12 months after surgery across all subscales. Although we cannot specify when in time these changes occurred, the observations are consistent with previous findings of vitality improving at 3 [15, 33] and 6 months [14] after TSS compared to preoperative status. Regarding individual change in the present study, more patients experienced a reduction in symptom severity than vice versa and, 12 months after TSS, a majority met the criterion for a normal level of fatigue. However, ~ 30% of the patients still met the criterion for severe fatigue.

For both cognition and fatigue, we found no indications of symptoms being more frequent or more severe 12 months after TSS. Whether surgery had an impact when symptoms did get more severe remains unclear. To determine the causes of symptoms would likely require a broader study scope, possibly including other factors such as hypothalamic function, hormonal status, and affective state. Further, since pituitary hormone deficiencies may be present before surgery for some patients and be new or resolve after surgery for others, subgroups tend to get small, which would require a larger study sample. In regards to the present study however, factors associated with symptoms might have been affected by surgery, but it seems that their net effect on symptoms was in general not more adverse after surgery than before. Consequences from surgery and subsequent care were seen primarily regarding fatigue, which might have contributed to the improvement seen for the cognitive domain of attention. However, the support for this is limited since only a non-significant indication of a relation between improvement in RBANS and MFI-20 total scores was found. Our previous cross-sectional study [16] showed a significant association between higher RBANS total index score and lower general fatigue subscale. Thus, the negative relation between cognitive performance and fatigue found in these studies was significant when analyzed based on absolute scores, but not when based on change in scores. One possible explanation might be, since fatigue improved whereas cognitive function remained stable, that the association was weakened because of different patterns of change.

Subgroup analysis showed that the improvement in attention did not remain significant for the subgroup with functioning pituitary adenoma. Similarly, in a study that included patients with somatotroph adenoma and NFPA, significant postoperative improvement in measurements of attention was seen only in the group of patients with NFPA [34]. In the present study, the groups of patients with functioning pituitary adenoma and NFPA both showed trends towards less fatigue after surgery on all MFI-20 dimensions and individual changes to a category of less fatigue were found among both groups. However, the reduction in fatigue was more pronounced among the patients with functioning pituitary adenoma, who reported worse fatigue compared to patients with NFPA before surgery.

Our study has several strengths and limitations. The longitudinal design of the present study comparing patients with their own baseline performances and employing parallel versions of cognitive tests administered by the same psychologist are strengths of this study. Although separate analyses were performed for the group with NFPA, the heterogeneity concerning tumor etiology is a limitation. Patterns of change for patients with functioning pituitary adenoma should be interpreted with caution due to the small size of this group. Further analyses by type of functioning pituitary adenoma were not possible. Although the primary aim of the study was to analyze change due to surgery, the influence of pituitary hormone deficiency and its replacement before and after surgery probably has a major impact on outcome and in explaining the patient-reported improvement in fatigue [35, 36].

Test performance before surgery might have been affected by distress and retest performance by practice effects. We have discussed the possible impact of distress previously [16]. Concerning practice effects, both generally higher mean scores at retest (~ 39 weeks, *n* = 40) [37] and no significant improvement in any index (~ 54 weeks, *n* = 445) [38] have been reported in studies using the same RBANS form on both occasions. In our study, patients might have become more familiar with the cognitive assessment at retest, but the use of alternate forms minimized any practice effects.

Categories of symptom severity were defined differently for cognition than for fatigue, so caution is required when comparing the occurrence of these respective problems. A single low index score did not necessarily entail a classification of cognitive impairment in this study. As noted by Binder and colleagues [39], an isolated low test score should be considered common in healthy people when using a battery of measures. Crawford and colleagues [40] have reported expected percentages for the normative population with low RBANS index scores. Categories of fatigue severity, in contrast, were based on the general fatigue subscale of MFI-20 and its 75th and 90th percentiles. Further, cognition was measured objectively and fatigue subjectively.

Even minor score changes may have entailed change in categories of symptoms if preoperative scores were close to a cutoff point. This may be of importance when comparing change based on categories of symptoms (with improvement/worsening ratio of ~ 2:1) with more negligible mean score change. Nonetheless, analyses based on categories of symptoms may add information regarding the frequency of symptoms of clinical severity and, perhaps more relevant for patients preparing for surgery, the frequency of changes in symptom severity after surgery.

## Conclusion

Our findings indicate that cognitive function remained stable 12 months after TSS, with significant improvement in attention, particularly in patients with NFPA. Fatigue levels decreased across multiple dimensions, with more pronounced improvements in patients with functioning pituitary adenoma. When individual change in severity of cognitive impairment or fatigue occurred, improvements were most common in absolute numbers. Overall, we could conclude that surgery in general does not negatively impact cognitive abilities and may enhance attention. Moreover, the reduction in fatigue could potentially lead to improved well-being and facilitate greater engagement in daily activities and rehabilitation.

## Electronic supplementary material

Below is the link to the electronic supplementary material.

Supplementary Material 1

## Data Availability

According to Swedish regulations (https://www.etikprovningsmyndigheten/), the datasets generated in this study cannot be made publicly available. Researchers may submit requests to David Krabbe (david.krabbe@gu.se) or to Head of Rehabilitation Medicine Research Group, Professor Katharina S. Sunnerhagen (ks.sunnerhagen@neuro.gu.se).
