# Supplementary material for: Cognitive function and fatigue before and after transsphenoidal surgery in patients with pituitary adenoma: a prospective study
Source: Pituitary. 2025 May 6;28(3):54. doi: 10.1007/s11102-025-01527-y (PMC12055867; doi:10.1007/s11102-025-01527-y)
Supplement: Supplementary file 1 — Supplementary Material 1 [file 11102_2025_1527_MOESM1_ESM.pdf]

Supplementary Information (SI)

"Cognitive function and fatigue before and after transsphenoidal surgery in patients with pituitary adenoma: a prospective study"

Pituitary

David Krabbe*, Tamar Abzhandadze, Thomas Skoglund, Tobias Hallén, Daniel S. Olsson, Victor Hantelius, Oskar Ragnarsson, Sofie Jakobsson, Gudmundur Johannsson, Katharina S. Sunnerhagen

***Corresponding author:** Department of Clinical Neuroscience, SU/Sahlgrenska, Blå stråket 7, plan 3, SE-413 45 Göteborg, Sweden; E-mail: david.krabbe@gu.se

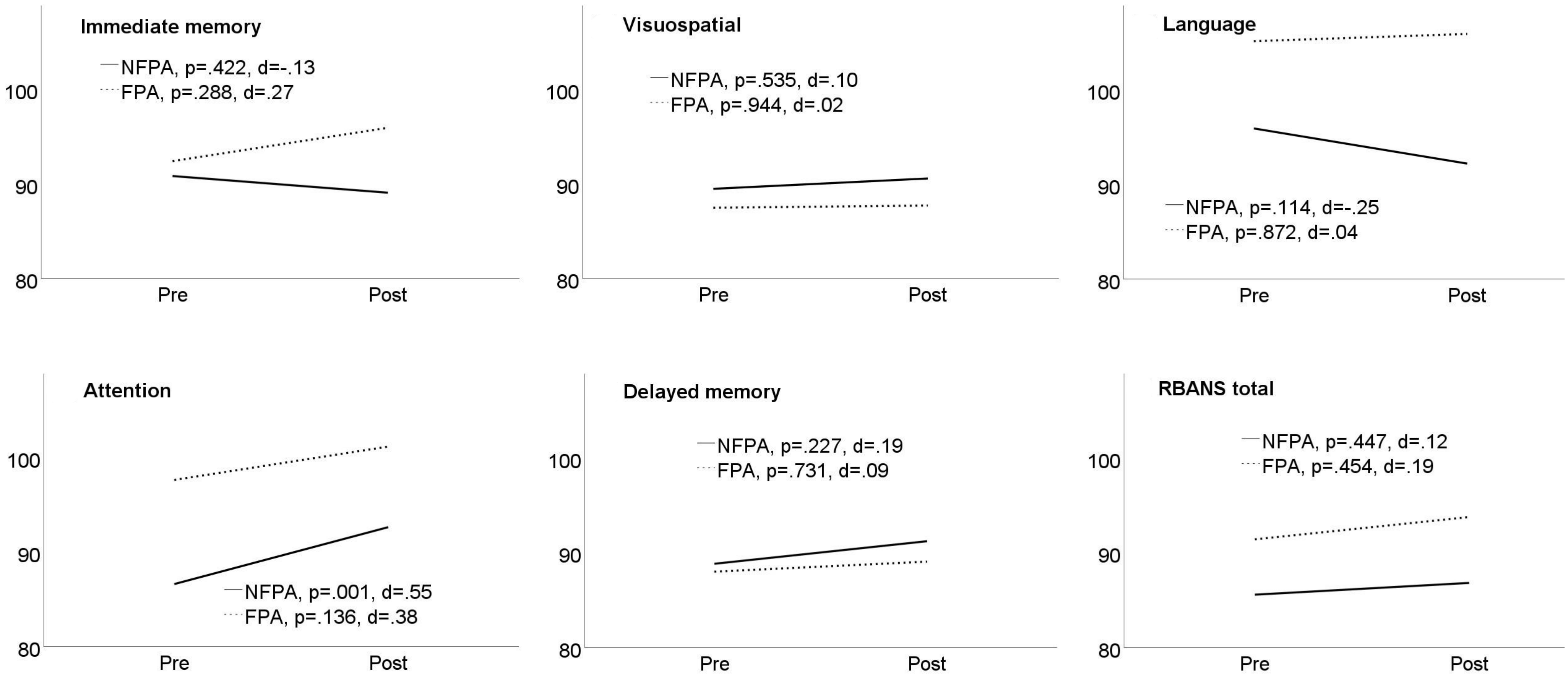

Supplementary Fig. 1. RBANS domain and total score before (pre) and 12 months after (post) surgery by NFPA (solid lines) and FPA (dotted lines) subgroups.

Paired samples *t*-tests and Cohen's effect size d were used for comparisons. *Abbreviations*: FPA, functioning pituitary adenoma; NFPA, non-functioning pituitary adenoma; RBANS, Repeatable Battery for the Assessment of Neuropsychological Status.

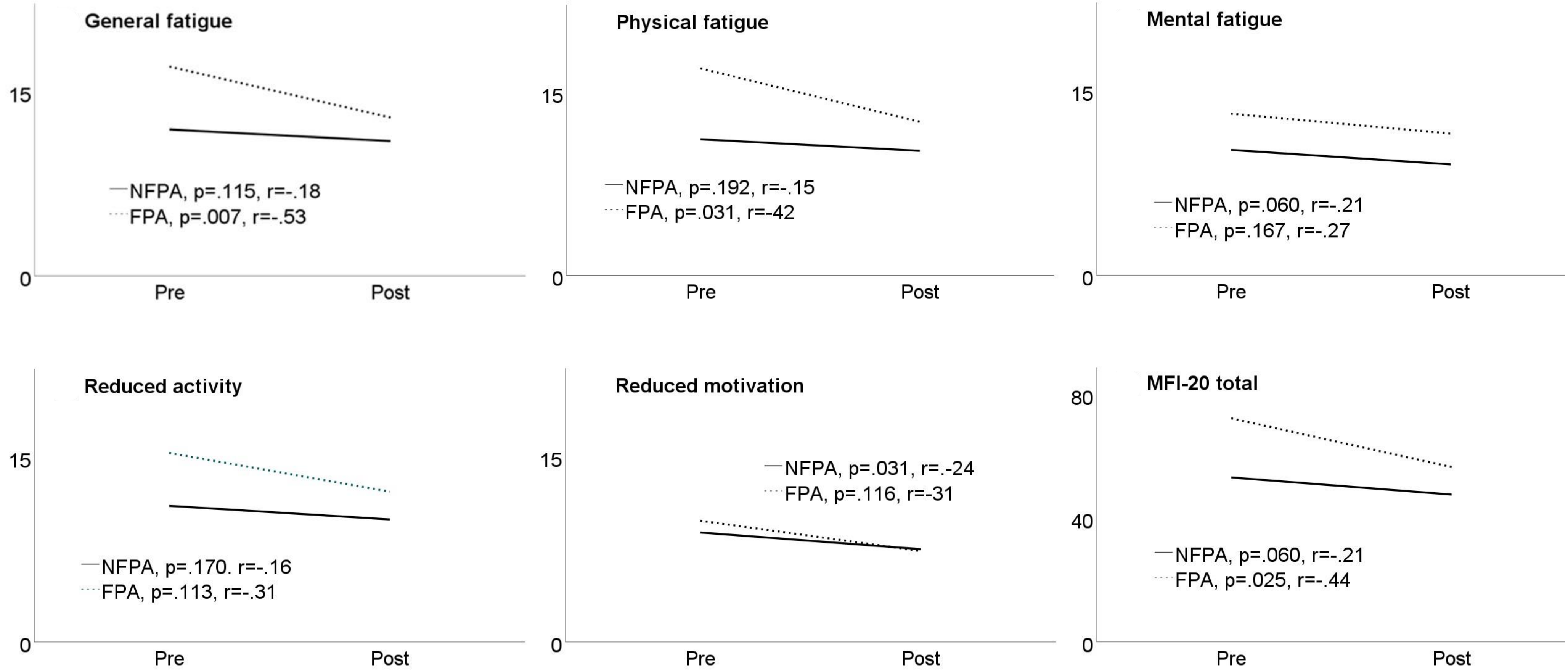

Supplementary Fig. 2. MFI-20 subscale and total score before (pre) and 12 months after (post) surgery by NFPA (solid lines) and FPA (dotted lines) subgroups.

Wilcoxon signed ranks tests and effect size r were used for comparisons. Data for MFI-20 before and/or after surgery was missing for 3 patients with NFPA (n = 39) and 4 patients with FPA (n = 13). Abbreviations: FPA, functioning pituitary adenoma; MFI-20, Multidimensional Fatigue Inventory-20; NFPA, non-functioning pituitary adenoma.

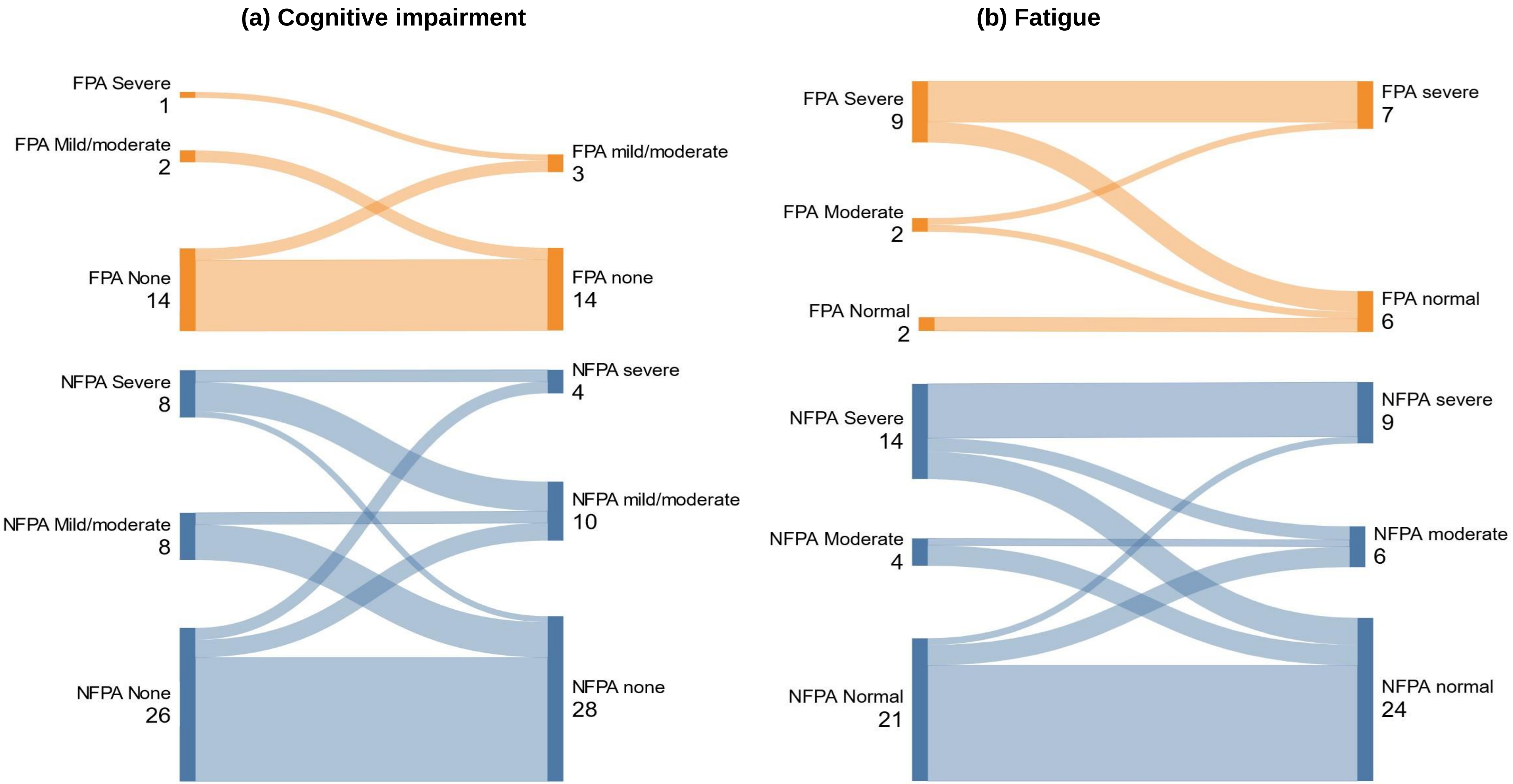

Supplementary Fig. 3. Level of symptoms before and 12 months after surgery and individual change for (a) cognition based on RBANS test results and (b) fatigue based on the general fatigue subscale of the MFI-20 by NFPA and FPA.

Abbreviations: RBANS, Repeatable Battery for the Assessment of Neuropsychological Status; MFI-20, Multidimensional Fatigue Inventory-20; FPA, functioning pituitary adenoma; NFPA, non-functioning pituitary adenoma.
